# Update of the organoprotective properties of xenon and argon: from bench to beside

**DOI:** 10.1186/s40635-020-0294-6

**Published:** 2020-02-24

**Authors:** Roehl Anna, Rossaint Rolf, Coburn Mark

**Affiliations:** Department of Anaesthesiology, Medical Faculty, RWTH Aachen University, Pauwelstrasse 30, 52072 Aachen, Germany

**Keywords:** Xenon, Argon, Cardioprotection, Nephroprotection, Neuroprotection, Clinical trials

## Abstract

The growth of the elderly population has led to an increase in patients with myocardial infarction and stroke (Wajngarten and Silva, Eur Cardiol 14: 111–115, 2019). Patients receiving treatment for ST-segment-elevation myocardial infarction (STEMI) highly profit from early reperfusion therapy under 3 h from the onset of symptoms. However, mortality from STEMI remains high due to the increase in age and comorbidities (Menees et al., N Engl J Med 369: 901–909, 2013). These factors also account for patients with acute ischaemic stroke. Reperfusion therapy has been established as the gold standard within the first 4 to 5 h after onset of symptoms (Powers et al., Stroke 49: e46-e110, 2018). Nonetheless, not all patients are eligible for reperfusion therapy. The same is true for traumatic brain injury patients. Due to the complexity of acute myocardial and central nervous injury (CNS), finding organ protective substances to improve the function of remote myocardium and the ischaemic penumbra of the brain is urgent. This narrative review focuses on the noble gases argon and xenon and their possible cardiac, renal and neuroprotectant properties in the elderly high-risk (surgical) population. The article will provide an overview of the latest experimental and clinical studies. It is beyond the scope of this review to give a detailed summary of the mechanistic understanding of organ protection by xenon and argon.

## Main text

Xenon was the first noble gas receiving thorough scientific interest starting in the field of decompression sickness of divers. In mice, the first experiments by Lawrence et al. described slightest signs of dizziness and movement disorders beginning from 60% of xenon [1]. The use of xenon as a general anaesthetic was first described in 1951 [2]. The low blood-gas and brain-blood coefficients, rapid induction and recovery from anaesthesia [3], almost no respiratory, hepatic or renal toxicity, stable haemodynamics, and effective organ protective properties in multiple animal models have made xenon a near “ideal” anaesthetic. Xenon has been shown to be safely applicable in patients, even in children [4–15]. Early clinical trials have shown the haemodynamic stability of xenon anaesthesia. The peripheral vascular resistance is increased. This increase leads to a higher mean arterial pressure, a lower heart frequency and a lower cardiac output compared to other inhalational anaesthetics or propofol [3, 6, 13, 14, 16–20]. A clinically relevant unpleasant side effect of xenon, despite its inhibition of 5-HT_3_-receptors, is the increase in postoperative nausea and vomiting (PONV) compared to propofol anaesthesia [5, 21–23].

Several narrative and systematic reviews have summarized the results from laboratory findings regarding the mechanistic understanding of the biological action of xenon [24–30].

An overview of the known molecular mechanisms of xenon is shown in Table 1.

**Table 1:** Molecular mechanism of xenon

Xenon				
Author	Effects revealed in human	Effects revealed in in vivo	Effects revealed in in vitro	Organprotection
Yakamura and Harris [31]			Non-competitive blockade of NMDA receptors and nACH (*N*-acetyl-choline receptors nACH)	
Li et al. [32]			mitoKATPchannel	Heart
Gruss et al. [33]		Activation of two-pore-domain K^+^channel (TREK-1)		Brain
Ma et al. [34]		Hypoxia inducible factor 1α↑ EPO ↑, VEGF↑ mTOR expression↑		Renal
Zhao et al. [35]			IGF-1↑, HIF 1α↑, NF-κB↓	Renal
Dinse et al. [36]			Blocks AMPA. receptor & kainate receptors	Brain
Bantel et al. [37]			K_ATP_ – Opener	Brain
Weber et al. [38], Weber et al. [39], Pravdic et al. [40]		Protein kinase C (PKC)-ε, EKR1/2 P38MAPK, HSP 27, JNK		Heart
Luo et al. [41]			PI3K Signaling	Brain
Banks et al. [42],			Competitive NMDA receptor inhibition	Brain
Harris et al. [43],			Competitive NMDA receptor inhibition Activation of TREK-1 channels	Brain
Franks et al. [44]			Inhibition of NMDA receptor	Brain

↑ upregulation; ↓ downregulation; ≈ no changes, blockade; *AMPA* a-amino-3-hydroxy-5-methyl-4-isoxazolole propionate, kainate; *Bcl-2* B cell lymphoma 2; *GABAA receptor* gamma-aminobutyric acid A receptor; *LPS* lipopolysaccharide; *ERK1/2* extracellular signal-regulated kinases 1/2; MEK1/2 = *MAPKK* mitogen-activated protein kinase; *mTOR* mammalian target of rapamycin; *Nrf2* nuclear factor (erythroid-derived 2)-like 3; *TLR* Toll-like receptor; *NF-κB* nuclear factor “kappa-light-chain-enhancer” of activated B cells

### Cardioprotection by xenon

In different experimental myocardial infarction models, xenon led to a relevant infarct size reduction as already known for isoflurane and sevoflurane [38, 39, 45–48]. Xenon given in pre-, peri- and postconditioning models has been shown to act on different effectors of the Survivor Activation Factor Enhancement (SAFE) pathway as well as the Reperfusion Injury Salvage Kinase (RISK) pathway [29, 38, 39, 46, 49, 50]. Animal studies have shown a better recovery of the stunned myocardium after ischaemia-reperfusion injury under xenon anaesthesia [49, 51]. A detailed description of the cardioprotective mechanism by xenon was gathered by Smit et al. [30]. Transferring results from one species to another is difficult, as inflammatory reactions in rodents differ from inflammatory reactions in pigs. However, the transfer from an acute myocardial pig infarction model [45, 46] to a chronic infarction model (4-week follow-up) in rats [52] underlined the cardioprotective properties of xenon. Following the demonstrated clinical cardiac stability and promising experimental infarct size reduction of xenon, an international, randomized clinical trial on the effect of xenon anaesthesia compared to sevoflurane and total intravenous anaesthesia for coronary artery bypass surgery was performed by Hofland et al. [53]. A total of 492 patients were randomized to receive either propofol, sevoflurane or xenon. As a primary outcome, the troponin I concentration was measured at 24 h postoperatively. For the first time, the cardioprotective properties of xenon were shown in patients. The troponin I reduction between xenon and propofol was significant. Sevoflurane showed the same extent of troponin I reduction. However, the impact of this observed reduced troponin I release (10 μg/l difference) on the postoperative outcome (30 days mortality, 1-year mortality) is difficult to determine. The translation of myocardial protection from the animal laboratory to cardiovascular surgery is quite difficult [54]. The ischaemia-reperfusion injury by cardioplegic solution and cardiac surgery itself commonly is quite small. The anaesthetic regimen (especially propofol) [55], surgical approach and cardioplegia/preservation may diminish the cardioprotective effect by xenon, as several substances, such as antihypertensive medication, similarly show cardioprotective properties [56, 57].

### Neuroprotection by xenon

Cardiac arrest due to ventricular fibrillation is the most life-threatening complication after myocardial infarction (1 out of 20 myocardial infarctions) [58]. Complete neurologic recovery is still the utmost therapy goal. Despite best efforts, up to 60% of the patients are neurologically or mentally disabled [59]. In this context, a substance with manifold organ protective properties would be of high interest.

The *N*-methyl-d-aspartate (NDMA) receptor is partly responsible for hypoxia-ischaemia-induced brain injury [60]. In vitro cardiac and neuroprotection of xenon is mediated by the inhibition of the excitatory NMDA receptor channels [24, 44, 61, 62]. In vitro models using glycine as a co-agonist of the NMDA receptor [42] as well as models of traumatic brain injury [43] could prove this mechanism of neuroprotection by xenon. This effect suggested that xenon might display a high neuroprotective potential. Another mechanism of possible neuroprotection by xenon has been shown by the modulation of two-pore potassium channels (TREK-1) [63]. Mild hypothermia has been clinically proven to be neuroprotective after cardiac arrest and perinatal asphyxia [64]. The first experiments in mice by Limatola et al. showed smaller cerebral infarct sizes when xenon (70%) was applied at 24 h before an ischaemic stroke. The upregulation of hypoxia inducible factor 1-α (HIF1-α) and anti-apoptotic B cell lymphoma (Bcl-2) was identified as possible mechanisms in xenon-treated animals [65]. Fries and colleagues were the first to show that xenon application at 60 min after CPR in pigs showed a better neurological outcome in the first three treatment days [66]. Reduced perivascular inflammation was observed in histopathologic examinations. Additional hypothermia reinforced the neuroprotective properties in this animal model [67]. This inflammation process after neurotrauma has a main impact on the extent of tissue damage and on the neuronal reparation process [68]. Neuroprotection was clinically observed as a significant improvement in the functional outcome of the animals and a further reduction in perivascular inflammation. The application of 30 or 45 Vol% xenon after 70 min of focal cerebral ischaemia [69] was only proven in combination with cerebral hypothermia to have a relevant impact on minimizing the neurologic outcome after 28 days in rats. These promising data were supported by Chakkarapani et al. investigating the effect of xenon and hypothermia in asphyxiated newborn pigs [70]. Xenon-treated animals showed significant improvements in histological and neurological improvement, although the animal survival only lasted up to 3 days. A feasibility study in preterm infants (*n* = 14) using xenon (50%) as an additional neuroprotectant compared to 72 h of hypothermia [12] showed no adverse effects. However, neuroprotective effects could not be demonstrated in this small cohort. Azzopardi et al. studied the effect of xenon in combination with hypothermia vs hypothermia alone in 92 infants with birth asphyxia [71]. Due to technical difficulties, the study was terminated prematurely. The sample size did not show significant neuroprotective properties in the different treatment groups. Another pilot study examined patients with out-of-hospital cardiac arrest (*n* = 36). The application of xenon in combination with hypothermia lasted for 24 h [72]. In addition to the clinically stable application in these high-risk patients, no relevant neurologic improvement could be shown. In a double-blind RCT, 110 comatose patients after cardiac arrest were randomized either to hypothermia alone or to hypothermia and xenon (68 Vol% inhaled) [73]. The diffusion tensor imaging revealed significantly less white matter damage measured by fractional anisotrophy in xenon-treated patients. This morphologic pattern did not lead to any neurologic clinical difference between the surviving patients in the two groups. This finding might be due to the small number of patients. Additionally, the time point of cooling varied relative to those showing neuroprotection by xenon in animal studies. The timing of xenon application in regard to neurologic injury (before, during or after the injury takes place) seems to have a relevant impact on the potential of the neuroprotective properties of xenon.

As mentioned above, the increase in the peripheral vascular resistance of xenon leads to an increase in cerebral blood flow [74, 75]. The only clinical trial (*n* = 13) using xenon (33 Vol%) in patients with head injury showed no cerebral decrease in perfusion due to increased blood flow by xenon anaesthesia, albeit increased intracranial pressure [76].

Another interesting aspect of neuroprotection is the potential reduction in postoperative cognitive deficit (POCD) by anaesthetic agents. The effect of anaesthetic agents and techniques on POCD is an ongoing matter of discussion. Several POCD risk factors that may be influenced interact with GABAerig signalling, such as other inhalational anaesthetics [77]. As xenon has no activity on GABA receptors [29], this gas might be a potential effector for minimizing POCD due to anaesthetic regimen. Two pilot studies in elective non-cardiac surgery (*n* = 38, *n* = 40) in patients (65–75 years) failed to show a decline in POCD [3, 78], probably due to the “too-healthy” collective. Bronco et al. demonstrated that xenon-treated patients (*n* = 60) had better cognitive recovery at 30 and 60 min after non-cardiac surgery compared to sevoflurane anaesthesia [19]. As patients after xenon anaesthesia showed faster emergence of anaesthesia than patients treated with sevoflurane, these results are not surprising. Interesting data regarding long-term outcomes are missing in this paper. An international multicentre phase II study (*n* = 256, xenon 60%) by Coburn et al. [8] performed a deeper examination of postoperative delirium (POD) in a high-risk population of elderly hip-fracture surgery patients up to 4 days after surgery. BIS monitoring was performed in all patients to exclude anaesthetic depth as a confounding factor. Xenon led to an overall POD reduction of 33% compared sevoflurane anaesthesia in this patient cohort. Due to the strict inclusion criteria, the incidence of POD was much smaller than expected from the literature in both groups. Therefore, the trial was underpowered to find statistical significance. The extent of injury due to anaesthesia leading to postoperative cognitive deficit has to be further elucidated, as the results are confounded by many other variables, such as intraoperative medication and haemodynamics, co-medication, postoperative care and duration of the procedure, e.g., the translation of positive results on neuroprotection by xenon from bench to bedside has not yet reached a clinical breakthrough. However, a recently published experimental study in mice by Campos-Pires et al. [79] provides a promise that neuroprotection by noble gases might still be worth for further research in this field. In an outstanding long-term experiment (20-month follow-up after traumatic brain injury), the cognitive function and survival rates were significantly improved by xenon in mice. Xenon treatment (70–75 Vol%) was initiated at 15 min after injury and lasted for 3 h. This experiment could show all the previously described beneficial properties of neuroprotection by xenon in one experiment: xenon treatment in this model reduced the loss of white matter and reactive astrogliosis known as long-term neuropathologic markers for traumatic brain injury. Especially in the CA1 and dentate gyros areas of the hypothalamus, xenon significantly preserved the number of intact neurons equivalent to the animals without traumatic brain injury. This neuropathological improvements in xenon-treated animals after TBI impeded late-onset hippocampus-dependent memory impairment.

### Nephroprotection by xenon

Acute kidney and liver injury after major cardiovascular surgery has an important impact on perioperative mortality. Hypoperfusion, inflammation and microembolism result in up to 30% of acute kidney failure (AKI) [80]. Experimental set-ups showed the relevant neuroprotective effects of xenon preconditioning in renal ischaemia-reperfusion injury [34]. As acute kidney injury is a risk factor after major cardiovascular surgery [80], a therapeutic agent with multiple organ protective properties would be of high interest. Ma et al. showed that 2 h of xenon anaesthesia (70 Vol%) before a renal ischaemia-reperfusion injury led to a 10-fold reduction in creatinine excretion in mice. Creatinine in xenon-treated animals normalized within 20 days. The major finding by Ma was the first proof of the upregulation of HIF-1α and the consecutive increase in erythropoietin (EPO) and vascular endothelial growth factor (VEGF) in xenon-treated animals. This erythropoietin increase is a downstream effector of the mammalian target of rapamycin (mTOR) pathway. Erythropoietin increase has a preconditioning and thereby organ protective effect in experimental ischaemia-reperfusion injury [81]. In a randomized controlled single centre trial, xenon (60 Vol%) was compared to isoflurane (1.2 Vol%) anaesthesia for nephroprotection in 46 patients who underwent partial nephrectomy with consecutive kidney ischaemia-reperfusion injury [82]. Xenon was equivalent to isoflurane anaesthesia, although not superior in regard to postoperative renal function in this relatively small cohort. This finding might be due to the relevant small ischaemia-reperfusion injury in this collective.

Apparently, xenon has been used for doping purposes due to its activation of HIF1-α [83] by Russian teams for endurance sports, such as biathlon, cross-country-skiing, e.g., during the Olympic winter games in Sotchi 2014 [84]. Since September 2014, xenon and argon have been added to the Prohibited List by the World Anti-Doping Agency (WADA) due to their activation of hypoxia tolerating factors, such as EPO and VEGF [85]. The minimal dose of xenon necessary to induce relevant HIf-1α is not yet known. The inhalation of subanaesthetic doses of xenon leads to a significant increase in HIf-1α followed by an up to 160% increase in EPO and VEGF [85]. Erythropoetin stimulation by xenon could be an interesting long-term benefit, for example, in patients with cerebral vasospasms. The injection of 30,000 IU of EPO for 3 days leads to a relevant reduction in the severity of cerebral vasospasm [86, 87]. By analysing blood, exhalation and urine samples of patients under xenon exposure, EPO elevation has been proven for 24 h [88–91]. In an RCT trial, 30 cardiac surgery patients received either xenon or sevoflurane. Stoppe et al. measured significantly elevated EPO concentrations in xenon-treated patients on the first postoperative day [91]. Even subanaesthetic doses of 30 Vol% xenon for 45 min lead to an increased EPO level in healthy volunteers [92]. The peak level was reached at 8 h after xenon exposure and remained elevated for up to 96 h. These are major differences in hypoxemia-induced erythropoietin stimulation, which only lasts up to 2 h [93]. This point might be of interest for further studies on the role of xenon as a possible neuroprotectant.

The use of xenon is licenced for adult anaesthesia in Europe [94]. Different manufacturers have developed anaesthesia circuits for xenon application, e.g., Air Liquide, EKU, Dräger. In addition to develop xenon-feasible anaesthesia circuits, the different measuring technologies and the calibration of flow sensors for xenon gas concentrations are challenging [95]. Commonly used thermal conductivity flow sensors in anaesthesia circuits have a measurement error of up to 10% of the actual xenon concentration due to the low heat conductance of xenon. Flow sensors based on ultrasound or laser refractometry show much better accuracy at lower costs. Incorrect measurement of xenon might lead to an unselected increase in xenon dosage, while xenon costs up to 20€/l [96]. In contrast, argon costs approximately 9c/l. Due to the recent changes in the Act on Medical Devices in European legislation, licenced anaesthesia circuits would have to renew their licence. In fact, there is no xenon-feasible anaesthesia circuit available at present, since the devices from Draeger, Air Liquide and EKU have been taken off the market. In small-animal experiments, fixed air-gas mixtures are used. The application of these manufactured air-gas mixtures would require up to four times more xenon than in closed or semi-closed anaesthesia circuits. Due to the high price of xenon as a consequence of the technical challenges of xenon extraction and constructing xenon anaesthesia circuits, the focus on organ protective noble gases has shifted towards argon. The main advantages of argon are its low cost, ease of transport and lack of anaesthetic properties under atmospheric pressure [97]. These advantages are of high value regarding any application in patients with stroke or any other cause of neuronal damage. The main focus in experimental settings of argon has been thus far on its neuroprotective properties.

### Neuroprotection by argon

Although the mechanisms of neuronal injury vary widely, the main goal of any neuroprotective agent will be to attenuate injury progression and diminish secondary injury due to hypoxia or ischaemia-reperfusion injury. Ideally, these mechanisms will lead to reduced neuronal and glial inflammation. These factors influence or even interrupt neuronal apoptosis, mitochondrial dysfunction and excitoxicity. Systematic reviews by Hoellig [98], Ulbrich [99] and Nespoli [100] have provided detailed information about the molecular pathways of neuroprotection mediated by argon (Table 2).

**Table 2:** Molecular mechanism of argon

Argon				
Author	Effects revealed in human	Effects revealed in in vivo	Effects revealed in in vitro	Organprotection
Loetscher et al. [101], Fahlenkamp et al. [102], Ulbrich et al. [103]			Activation of MEK-ERK 1/2pathway	Brain
Abraini et al. [104]		Binding to GABA_A_-receptor		Brain
Fahlenkamp et al. [105]		↑ IL-1beta, ↑IL-6, ↑iNOS, ↑TGF-beta,		Brain
Zhuang et al. [106]		↑ Bcl-2		Brain
Ulbrich et al. [107]			Inhibition TLR 2/4	Brain
Ulbrich et al. [103]			↑ERK1/2,↑Heme-oxygenase-1	Brain
Zhao et al. [108]			PI3K Signaling, ↑Nrf2	Brain

↑ upregulation; ↓ downregulation; ≈ no changes, blockade; *AMPA* a-amino-3-hydroxy-5-methyl-4-isoxazolole propionate, kainate; *Bcl-2* B cell lymphoma 2; *GABAA receptor* gamma-aminobutyric acid A receptor; *LPS* lipopolysaccharide; *ERK1/2* extracellular signal-regulated kinases 1/2; MEK1/2 = *MAPKK* mitogen-activated protein kinase; *mTOR* mammalian target of rapamycin; *Nrf2* nuclear factor (erythroid-derived 2)-like 3; *TLR* Toll-like receptor; *NF-κB* nuclear factor “kappa-light-chain-enhancer” of activated B cells

Different in vitro models of argon application in organotypic hippocampal slices proved the neuroprotective properties of argon at 1 h and 2 h after neurologic injury [101]. The best effect in vivo was shown for a concentration of 50 Vol% inhaled argon [101]. Recently, Koziakova et al. compared the neuroprotective effects of xenon and argon in the same model of organotypic hippocampal brain slices [109]. Both substances impaired the neurologic injury to the same extent. Additionally, the neuroprotective effect of xenon was abolished by glycine. This finding underlines the neuroprotective properties of xenon mediated via the inhibition of the NMDA receptor glycine site. Neuroprotection by argon was not impaired by glycine. The detailed pathway of neuroprotection by argon still has to be elucidated.

Models of global cerebral ischaemia with the inhalation of 70 Vol% argon for 1 h after cardiopulmonary resuscitation (CPR) in rats underline the neuroprotective properties of argon in vivo. Histopathological damage in the cortex and hippocampal region was reduced, resulting in an improved functional neurological outcome up to 3 days after injury. This improvement was even shown by applying argon 1 delayed hour after CPR [110, 111]. Following these promising results, argon was added to mild hypothermia in the same model. This addition is because hypothermia is currently the only proven neuroprotective method in humans [64]. Argon added to hypothermia compared to animals treated with hypothermia alone, which showed worse neurologic outcomes and the deterioration of histopathologic results. These results need to be further explored. Other models tested for the neuroprotective effects of argon are experimental stroke models or models of experimental subarachnoid haemorrhage. Applying argon (50 Vol%) either during ischaemia or after reperfusion (24 Vol%) for 3 h in a transient middle cerebral artery occlusion model reduced the infarct size [112, 113]. These experiments did not lead to an improvement in neurologic outcome. Recently, Ma et al. [114] proved that argon (70 Vol%) applied for 24 h in a MCAO model relevantly improved the neurologic outcome and reduced the final infarct volumes.

Inflammation plays a major role after stroke by aggravating neuronal damage in the penumbra [86, 115]. Fahlenkamp et al. showed that argon reduces LPS-induced inflammation in microglia cell cultures [102]. Fore a deeper mechanistic understanding of neuroprotection by argon models of apoptosis in human neuroblastoma cells and ischaemia reperfusion were established by Ulbrich et al. [99, 107, 116]. Ulbrich et al. identified the inhibition of Toll-like receptors (TLR2/TLR4) and the activation of the NF-κB pathway by argon as relevant regarding the reduction in microglial inflammation. Initial microglial activation in ischaemic stroke is highly attributed to blood-brain barrier disruption [115]. Due to these results, the focus of neuroprotection by argon is on the ischaemic boundary zone. An experimental MCAO model in rats by Liu et al. [117] did not have any impact on the infarct volume. However, Liu showed that argon has an impact on microglial inflammation in vivo [117].. Argon (50 Vol%) application at 3 h after the onset of stroke and at 1 h after reperfusion led to a decrease in microglial inflammation in the ischaemic border zone. The anti-inflammatory M2 phenotype of microglia was upregulated in this model. This positive influence on neuronal recovery in the ischaemic boundary zone at 7 days after reperfusion resulted in a significant reduction in neurologic deficit. The microglia activation by ischaemia-reperfusion injury leads to an increase in oxidative stress, inflammation and apoptosis [118]. Ma et al. applied argon (70%) for 24 h in a permanent MCAO model in rats onset 2 h after ischaemia [114]. In this model, infarct volume and 7-day mortality were not significantly reduced in argon-treated animals. However, surviving animals showed a significant improvement in neurologic outcome. Reperfusion activates microglia. The activation of microglia is associated with oxidative stress, inflammation and apoptosis. These three factors have been shown to have a deleterious impact on ischaemic stroke [115, 119]. Recent evidence shows a biphasic role for microglia. Therefore, a deeper investigation of the long-term effect of argon on microglial activation should be performed. Perhaps this finding could explain the different infarct-size reduction effects of argon depending on the time point of argon application. If there is a long-term reduction in microglial activation by argon, this effect might influence the shift from a detrimental to a beneficial microglial phenotype [115].

In kidney transplantation, ischaemia-reperfusion injury is predictable. Three different experimental models investigated the renoprotective properties of argon. In rats, argon-saturated storage solution significantly improved renal function, even up to 14 days posttransplantation [120]. In a pig model of heterotropic renal autotransplantation, saturation of the extracellular preservation solution with argon showed acute and long-term (21 days) positive impacts on ischaemia-reperfusion injury [121]. Meanwhile, postconditioning with argon for impairment of renal ischaemia-reperfusion injury (IRI) does not improve kidney graft function in a similar porcine autotransplantation model [122]. Argon (70 Vol%) applied before aortic cross clamping attenuated ischaemia-reperfusion injury to multiple organs in a rabbit model [123]. It needs further studies to clarify the role of argon on the attenuation of the ischaemia-reperfusion injury leading to a long-term improvement in kidney function.

### Argon in human patients

Clinically, fixed argon-oxygen concentrations are used as an indicator for cerebrovascular CO_2_-reactivity of cerebral blood flow [124, 125]. The growing interest in possible clinical settings led to a deeper look at this established argon inhalation in human physiology as a proof of principle. In 30 patients prior to cardiac surgery, argon was applied for 15 min only. Inhalation of argon did not increase cerebral perfusion, global oxygen consumption or glucose metabolism in this cohort. To apply argon for human use, a feasible application method needs to be developed. In a short correspondence by Cucino et al. [126], a modified anaesthesia circuit (Bellavista 1000, IMT Medical, Buchs) was used to deliver 70% argon and 30% oxygen in pigs regarding the promising argon concentration for neuroprotection. This is a promising opportunity for standardizing argon while ventilating patients under critical conditions. Fixed argon-oxygen mixtures used in animal experiments will be less comfortable for use in critically ill patients. As we have no clinical experience with argon application in humans, new insights into the pulmonary vascular tone in rats and humans have been reported by Suleiman et al. [127]. These cell culture experiments present the first evidence that unlike xenon, argon will not be irritating to the airways. Additionally, it could be shown that argon decreases the pulmonary vessel tone due to GABA receptor activation. This effect might be of high value if argon-treated patients show relevant comorbidities, such as right ventricular failure or pulmonary hypertension.

### Conclusions

One of the main questions remains. Why do we still lack to transfer these promising results of experimental neuroprotection by noble gases to clinical settings? There is now a wide mechanistical understanding of organprotection by xenon. The clinical robustness of xenon anaesthesia has been proven. These positive findings highly enforce further interest in organprotection by noble gases. The high price of xenon and the lack of available xenon-capable ventilators will impede further clinical trials on xenon. Therefore, effort should be put into developing ventilators feasible for xenon or argon anaesthesia. This development will take some time due to manufacturing cycles and legislative requirements. Therefore, it might be tempting to use fixed air-argon concentrations for the first clinical trials, although these methods also have to be accredited. Dingley et al. developed a portable delivery device for xenon application children after perinatal asphyxia [128]. By this device, xenon application was feasible within 5 h after birth. This device is not yet commercially available.

One main commonality in all experimental organ protective studies is that no propofol is used in the animal setting for the induction of anaesthesia. Most inhalation agents or the intraperitoneal application of thiopental are used for anaesthesia induction in animals. If propofol itself exceeds cardioprotective properties or adversely attenuates protection, then the experiment is not yet conclusive due to the available data. Indeed, propofol is ubiquitous for the induction of human anaesthesia worldwide. Recently, research focused on the impact of propofol on promoting/inhibiting remote-ischaemic preconditioning, anaesthetic organ protection, neuroprotection by reducing/increasing POD, etc. [55, 129–133].

Consensus papers have been published for planning preclinical and human trials in myocardial per- and postconditioning [56] for stroke therapy (Stroke Therapy Acute Industry Roundtable STAIR recommendations) [92] and for traumatic brain injury [134, 138]. STAIR recommendations first published in 1999 have not been implemented in many animal models until 2015, as reviewed by Thomas et al. [135]. Most likely, consensus studies on neuroprotection by noble gases would help to focus on comparable protocols in experimental studies, for example, comparable anaesthesia induction, maintenance and pain therapy. Currently, there is a wide mechanistic understanding of how argon promotes organprotection, mostly centred on insights into neuroprotection and in acute to short-term models (less than 7 day survival). These results are highly promising. Perhaps long-term models, such as those described for traumatic brain injury by Campos-Pires [79], could enforce the upscaling from short-term experimental settings to human trials.

One main difference between experimental and clinical trials with xenon for neuroprotection after resuscitation or neonatal asphyxia is the time window of xenon application. In animal studies, the time window ranges from 1 to 5 h [69], over 3 h [136] up to 4 h [137] after the timepoint of defined neuronal injury. In the human, Xe-Hypotheca trial (xenon application combined with hypothermia after out of hospital arrest) started 5 h after the incident what comes close to the therapeutic time-windows identified in animal trials [52]. Xenon treatment lasted up to 72 h and resulted in a significantly reduced release in troponin T. This pilot trial would be worth giving xenon a trial in bigger randomized clinical trials.

In contrast, the Toby-XE trial (xenon application starting within the first 6 h after onset of neonatal hypoxic-ischemic brain injury) could not prove any neuroprotective effects [71]. Both human trials showed the feasibility of applying xenon in emergency situations in patients with a relevant cell injury. The time delay in xenon application compared to experimental settings seems to have a relevant impact on the potency of neuroprotection.0

In preclinical trials, the organ protective effects have to be investigated in senescent, co-medicated and animals of different sexes. Planning clinical translation should focus on trial efficacy, robustness, clinical relevance, therapeutic window, interactions with other standard therapies (revascularization, hypothermia), physiologic monitoring, outcome measures, sex and age, co-medication differences, etc. Additionally, electronical data recording for any preclinical and clinical trials might elucidate further aspects of neuroprotection by noble gases using big data analysis. At least standardized charts leading to a database for experimental set-ups could be worth considering.

## Data Availability

Not applicable

## Abbreviations

AKI: Acute kidney failure
Bcl-2: B cell lymphoma
BIS-index: Bispectral index
CPR: Cardiopulmonary resuscitation
GABA: Gamma-aminobutyric acid
HIF1-α: Hypoxia inducible factor 1-α
IRI: Ischaemia-reperfusion injury
LPS: Lipopolysaccharide
NMDA: *N*-methyl-d-aspartate
POD: Postoperative cognitive deficit
PONV: Postoperative nausea and vomiting
RCT: Randomized controlled trial
STAIR: Stroke Therapy Acute Industry Roundtable recommendations
STEMI: ST-segment-elevation myocardial infarction
TREK-1: Two-pore potassium channel
VEGF: Vascular endothelial growth factor
WADA: World Anti-Doping Agency

## References

[1] Lawrence JH, Loomis WF, Tobias CA, Turpin FH (1946). Preliminary observations on the narcotic effect of xenon with a review of values for solubilities of gases in water and oils. J Physiol.

[2] Cullen SC, Gross EG (1951). The anesthetic properties of xenon in animals and human beings, with additional observations on krypton. Science.

[3] Coburn M, Baumert JH, Roertgen D, Thiel V, Fries M, Hein M, Kunitz O, Fimm B, Rossaint R (2007). Emergence and early cognitive function in the elderly after xenon or desflurane anaesthesia: a double-blinded randomized controlled trial. Br J Anaesth.

[4] Xia Y, Fang H, Xu J, Jia C, Tao G, Yu B (2018). Clinical efficacy of xenon versus propofol: a systematic review and meta-analysis. Medicine (Baltimore).

[5] Coburn M, Kunitz O, Apfel CC, Hein M, Fries M, Rossaint R (2008). Incidence of postoperative nausea and emetic episodes after xenon anaesthesia compared with propofol-based anaesthesia. Br J Anaesth.

[6] Coburn M, Kunitz O, Baumert JH, Hecker K, Haaf S, Zuhlsdorff A, Beeker T, Rossaint R (2005). Randomized controlled trial of the haemodynamic and recovery effects of xenon or propofol anaesthesia. Br J Anaesth.

[7] Coburn M, Kunitz O, Baumert JH, Hecker K, Rossaint R (2005). Patients’ self-evaluation after 4-12 weeks following xenon or propofol anaesthesia: a comparison. Eur J Anaesthesiol.

[8] Coburn M, Sanders RD, Maze M, Nguyen-Pascal ML, Rex S, Garrigues B, Carbonell JA, Garcia-Perez ML, Stevanovic A, Kienbaum P, Neukirchen M, Schaefer MS, Borghi B, van Oven H, Tognu A, Al Tmimi L, Eyrolle L, Langeron O, Capdevila X, Arnold GM, Schaller M, Rossaint R, Investigators HS (2018). The hip fracture surgery in elderly patients (HIPELD) study to evaluate xenon anaesthesia for the prevention of postoperative delirium: a multicentre, randomized clinical trial. Br J Anaesth.

[9] Al Tmimi L, Devroe S, Dewinter G, Van de Velde M, Poortmans G, Meyns B, Meuris B, Coburn M, Rex S (2017). Xenon as an adjuvant to propofol anesthesia in patients undergoing off-pump coronary artery bypass graft surgery: a pragmatic randomized controlled clinical trial. Anesth Analg.

[10] Baumert JH, Hein M, Hecker KE, Satlow S, Neef P, Rossaint R (2008). Xenon or propofol anaesthesia for patients at cardiovascular risk in non-cardiac surgery. Br J Anaesth.

[11] Baumert JH, Hein M, Hecker KE, Satlow S, Schnoor J, Rossaint R (2007). Autonomic cardiac control with xenon anaesthesia in patients at cardiovascular risk. Br J Anaesth.

[12] Dingley J, Tooley J, Liu X, Scull-Brown E, Elstad M, Chakkarapani E, Sabir H, Thoresen M (2014). Xenon ventilation during therapeutic hypothermia in neonatal encephalopathy: a feasibility study. Pediatrics.

[13] Goto T, Hanne P, Ishiguro Y, Ichinose F, Niimi Y, Morita S (2004). Cardiovascular effects of xenon and nitrous oxide in patients during fentanyl-midazolam anaesthesia. Anaesthesia.

[14] Wappler F, Rossaint R, Baumert J, Scholz J, Tonner PH, van Aken H, Berendes E, Klein J, Gommers D, Hammerle A, Franke A, Hofmann T, Schulte Esch J, Xenon Multicenter Study Research G (2007). Multicenter randomized comparison of xenon and isoflurane on left ventricular function in patients undergoing elective surgery. Anesthesiology.

[15] Devroe S, Meeusen R, Gewillig M, Cools B, Poesen K, Sanders R, Rex S (2017). Xenon as an adjuvant to sevoflurane anesthesia in children younger than 4 years of age, undergoing interventional or diagnostic cardiac catheterization: a randomized controlled clinical trial. Paediatr Anaesth.

[16] Rossaint R, Reyle-Hahn M, Schulte Am Esch J, Scholz J, Scherpereel P, Vallet B, Giunta F, Del Turco M, Erdmann W, Tenbrinck R, Hammerle AF, Nagele P, Xenon Study G (2003). Multicenter randomized comparison of the efficacy and safety of xenon and isoflurane in patients undergoing elective surgery. Anesthesiology.

[17] Baumert JH, Hecker KE, Hein M, Reyle-Hahn M, Horn NA, Rossaint R (2005). Effects of xenon anaesthesia on the circulatory response to hypoventilation. Br J Anaesth.

[18] Baumert JH, Falter F, Eletr D, Hecker KE, Reyle-Hahn M, Rossaint R (2005). Xenon anaesthesia may preserve cardiovascular function in patients with heart failure. Acta Anaesthesiol Scand.

[19] Bronco A, Ingelmo PM, Aprigliano M, Turella M, Sahillioglu E, Bucciero M, Somaini M, Fumagalli R (2010). Xenon anaesthesia produces better early postoperative cognitive recovery than sevoflurane anaesthesia. Eur J Anaesthesiol.

[20] Al Tmimi L, Van Hemelrijck J, Van de Velde M, Sergeant P, Meyns B, Missant C, Jochmans I, Poesen K, Coburn M, Rex S (2015). Xenon anaesthesia for patients undergoing off-pump coronary artery bypass graft surgery: a prospective randomized controlled pilot trial. Br J Anaesth.

[21] Schaefer MS, Apfel CC, Sachs HJ, Stuttmann R, Bein B, Tonner PH, Hein M, Neukirchen M, Reyle-Hahn M, Kienbaum P (2015). Predictors for postoperative nausea and vomiting after xenon-based anaesthesia. Br J Anaesth.

[22] Fahlenkamp AV, Stoppe C, Cremer J, Biener IA, Peters D, Leuchter R, Eisert A, Apfel CC, Rossaint R, Coburn M (2016). Nausea and vomiting following balanced xenon anesthesia compared to sevoflurane: a post-hoc explorative analysis of a randomized controlled trial. PLoS One.

[23] Suzuki T, Ueta K, Sugimoto M, Uchida I, Mashimo T (2003). Nitrous oxide and xenon inhibit the human (alpha 7)5 nicotinic acetylcholine receptor expressed in Xenopus oocyte. Anesth Analg.

[24] Dickinson R, Franks NP (2010). Bench-to-bedside review: molecular pharmacology and clinical use of inert gases in anesthesia and neuroprotection. Crit Care.

[25] De Deken J, Rex S, Monbaliu D, Pirenne J, Jochmans I (2016). The efficacy of noble gases in the attenuation of ischemia reperfusion injury: a systematic review and meta-analyses. Crit Care Med.

[26] Fahlenkamp AV, Rossaint R, Coburn M (2015). Neuroprotection by noble gases: new developments and insights. Anaesthesist.

[27] Law LS, Lo EA, Gan TJ (2016). Xenon anesthesia: a systematic review and meta-analysis of randomized controlled trials. Anesth Analg.

[28] Mangus DB, Huang L, Applegate PM, Gatling JW, Zhang J, Applegate RL (2014). A systematic review of neuroprotective strategies after cardiac arrest: from bench to bedside (part I - protection via specific pathways). Med Gas Res.

[29] Preckel B, Weber NC, Sanders RD, Maze M, Schlack W (2006). Molecular mechanisms transducing the anesthetic, analgesic, and organ-protective actions of xenon. Anesthesiology.

[30] Smit KF, Weber NC, Hollmann MW, Preckel B (2015). Noble gases as cardioprotectants - translatability and mechanism. Br J Pharmacol.

[31] Yamakura T, Harris RA (2000). Effects of gaseous anesthetics nitrous oxide and xenon on ligand-gated ion channels. Comparison Isoflurane Ethanol Anesthesiol.

[32] Li Q, Lian C, Zhou R, Li T, Xiang X, Liu B (2013). Pretreatment with xenon protected immature rabbit heart from ischaemia/reperfusion injury by opening of the mitoKATP channel. Heart Lung Circ.

[33] Grusser L, Blaumeiser-Debarry R, Krings M, Kremer B, Hollig A, Rossaint R, Coburn M (2017). Argon attenuates the emergence of secondary injury after traumatic brain injury within a 2-hour incubation period compared to desflurane: an in vitro study. Med Gas Res.

[34] Ma D, Lim T, Xu J, Tang H, Wan Y, Zhao H, Hossain M, Maxwell PH, Maze M (2009). Xenon preconditioning protects against renal ischemic-reperfusion injury via HIF-1alpha activation. J Am Soc Nephrol.

[35] Zhao H, Luo X, Zhou Z, Liu J, Tralau-Stewart C, George AJ, Ma D (2014). Early treatment with xenon protects against the cold ischemia associated with chronic allograft nephropathy in rats. Kidney Int.

[36] Dinse A, Fohr KJ, Georgieff M, Beyer C, Bulling A, Weigt HU (2005). Xenon reduces glutamate-, AMPA-, and kainate-induced membrane currents in cortical neurones. Br J Anaesth.

[37] Bantel C, Maze M, Trapp S (2009). Neuronal preconditioning by inhalational anesthetics: evidence for the role of plasmalemmal adenosine triphosphate-sensitive potassium channels. Anesthesiology.

[38] Weber NC, Stursberg J, Wirthle NM, Toma O, Schlack W, Preckel B (2006). Xenon preconditioning differently regulates p44/42 MAPK (ERK 1/2) and p46/54 MAPK (JNK 1/2 and 3) in vivo. Br J Anaesth.

[39] Weber NC, Toma O, Wolter JI, Wirthle NM, Schlack W, Preckel B (2005). Mechanisms of xenon- and isoflurane-induced preconditioning - a potential link to the cytoskeleton via the MAPKAPK-2/HSP27 pathway. Br J Pharmacol.

[40] Pravdic D, Sedlic F, Mio Y, Vladic N, Bienengraeber M, Bosnjak ZJ (2009). Anesthetic-induced preconditioning delays opening of mitochondrial permeability transition pore via protein kinase C-epsilon-mediated pathway. Anesthesiology.

[41] Luo Y, Ma D, Ieong E, Sanders RD, Yu B, Hossain M, Maze M (2008). Xenon and sevoflurane protect against brain injury in a neonatal asphyxia model. Anesthesiology.

[42] Banks P, Franks NP, Dickinson R (2010). Competitive inhibition at the glycine site of the N-methyl-D-aspartate receptor mediates xenon neuroprotection against hypoxia-ischemia. Anesthesiology.

[43] Harris K, Armstrong SP, Campos-Pires R, Kiru L, Franks NP, Dickinson R (2013). Neuroprotection against traumatic brain injury by xenon, but not argon, is mediated by inhibition at the N-methyl-D-aspartate receptor glycine site. Anesthesiology.

[44] Franks NP, Dickinson R, de Sousa SL, Hall AC, Lieb WR (1998). How does xenon produce anaesthesia?. Nature.

[45] Baumert JH, Hein M, Gerets C, Baltus T, Hecker KE, Rossaint R (2007). The effect of xenon anesthesia on the size of experimental myocardial infarction. Anesth Analg.

[46] Hein M, Roehl AB, Baumert JH, Bantes B, Bleilevens C, Bernstein N, Steendijk P, Rossaint R (2008). Establishment of a porcine right ventricular infarction model for cardioprotective actions of xenon and isoflurane. Acta Anaesthesiol Scand.

[47] Roehl AB, Funcke S, Becker MM, Goetzenich A, Bleilevens C, Rossaint R, Steendijk P, Hein M (2013). Xenon and isoflurane reduce left ventricular remodeling after myocardial infarction in the rat. Anesthesiology.

[48] Obal D, Dettwiler S, Favoccia C, Rascher K, Preckel B, Schlack W (2006). Effect of sevoflurane preconditioning on ischaemia/reperfusion injury in the rat kidney in vivo. Eur J Anaesthesiol.

[49] Hartlage MA, Berendes E, Van Aken H, Fobker M, Theisen M, Weber TP (2004). Xenon improves recovery from myocardial stunning in chronically instrumented dogs. Anesth Analg.

[50] Breuer T, Emontzpohl C, Coburn M, Benstoem C, Rossaint R, Marx G, Schalte G, Bernhagen J, Bruells CS, Goetzenich A, Stoppe C (2015). Xenon triggers pro-inflammatory effects and suppresses the anti-inflammatory response compared to sevoflurane in patients undergoing cardiac surgery. Crit Care.

[51] Preckel B, Mullenheim J, Moloschavij A, Thamer V, Schlack W (2000). Xenon administration during early reperfusion reduces infarct size after regional ischemia in the rabbit heart in vivo. Anesth Analg.

[52] Arola O, Saraste A, Laitio R, Airaksinen J, Hynninen M, Backlund M, Ylikoski E, Wennervirta J, Pietila M, Roine RO, Harjola VP, Niiranen J, Korpi K, Varpula M, Scheinin H, Maze M, Vahlberg T, Laitio T, Xe HSG (2017). Inhaled xenon attenuates myocardial damage in comatose survivors of out-of-hospital cardiac arrest: the Xe-Hypotheca trial. J Am Coll Cardiol.

[53] Hofland J, Ouattara A, Fellahi JL, Gruenewald M, Hazebroucq J, Ecoffey C, Joseph P, Heringlake M, Steib A, Coburn M, Amour J, Rozec B, Liefde I, Meybohm P, Preckel B, Hanouz JL, Tritapepe L, Tonner P, Benhaoua H, Roesner JP, Bein B, Hanouz L, Tenbrinck R, Bogers A, Mik BG, Coiffic A, Renner J, Steinfath M, Francksen H, Broch O, Haneya A, Schaller M, Guinet P, Daviet L, Brianchon C, Rosier S, Lehot JJ, Paarmann H, Schon J, Hanke T, Ettel J, Olsson S, Klotz S, Samet A, Laurinenas G, Thibaud A, Cristinar M, Collanges O, Levy F, Rossaint R, Stevanovic A, Schaelte G, Stoppe C, Hamou NA, Hariri S, Quessard A, Carillion A, Morin H, Silleran J, Robert D, Crouzet AS, Zacharowski K, Reyher C, Iken S, Weber NC, Hollmann M, Eberl S, Carriero G, Collacchi D, Di Persio A, Fourcade O, Bergt S, Alms A, Xenon CSG (2017). Effect of xenon anesthesia compared to sevoflurane and total intravenous anesthesia for coronary artery bypass graft surgery on postoperative cardiac troponin release: an international, multicenter, phase 3, single-blinded, randomized noninferiority trial. Anesthesiology.

[54] Heusch G, Rassaf T (2016). Time to give up on cardioprotection? A critical appraisal of clinical studies on ischemic pre-, post-, and remote conditioning. Circ Res.

[55] Kottenberg E, Thielmann M, Bergmann L, Heine T, Jakob H, Heusch G, Peters J (2012). Protection by remote ischemic preconditioning during coronary artery bypass graft surgery with isoflurane but not propofol - a clinical trial. Acta Anaesthesiol Scand.

[56] Bell RM, Botker HE, Carr RD, Davidson SM, Downey JM, Dutka DP, Heusch G, Ibanez B, Macallister R, Stoppe C, Ovize M, Redington A, Walker JM, Yellon DM (2016). 9th Hatter Biannual Meeting: position document on ischaemia/reperfusion injury, conditioning and the ten commandments of cardioprotection. Basic Res Cardiol.

[57] Ferdinandy P, Hausenloy DJ, Heusch G, Baxter GF, Schulz R (2014). Interaction of risk factors, comorbidities, and comedications with ischemia/reperfusion injury and cardioprotection by preconditioning, postconditioning, and remote conditioning. Pharmacol Rev.

[58] Karam N, Bataille S, Marijon E, Tafflet M, Benamer H, Caussin C, Garot P, Juliard JM, Pires V, Boche T, Dupas F, Le Bail G, Lamhaut L, Simon B, Allonneau A, Mapouata M, Loyeau A, Empana JP, Lapostolle F, Spaulding C, Jouven X, Lambert Y, e MSI (2019). Incidence, mortality, and outcome-predictors of sudden cardiac arrest complicating myocardial infarction prior to hospital admission. Circ Cardiovasc Interv.

[59] Wenzel V, Krismer AC, Arntz HR, Sitter H, Stadlbauer KH, Lindner KH, European Resuscitation Council Vasopressor during Cardiopulmonary Resuscitation Study G (2004). A comparison of vasopressin and epinephrine for out-of-hospital cardiopulmonary resuscitation. N Engl J Med.

[60] Lipton SA, Rosenberg PA (1994). Excitatory amino acids as a final common pathway for neurologic disorders. N Engl J Med.

[61] White IL, Franks NP, Dickinson R (2005). Effects of isoflurane and xenon on Ba2+−currents mediated by N-type calcium channels. Br J Anaesth.

[62] Dickinson R, Peterson BK, Banks P, Simillis C, Martin JC, Valenzuela CA, Maze M, Franks NP (2007). Competitive inhibition at the glycine site of the N-methyl-D-aspartate receptor by the anesthetics xenon and isoflurane: evidence from molecular modeling and electrophysiology. Anesthesiology.

[63] Gruss M, Bushell TJ, Bright DP, Lieb WR, Mathie A, Franks NP (2004). Two-pore-domain K+ channels are a novel target for the anesthetic gases xenon, nitrous oxide, and cyclopropane. Mol Pharmacol.

[64] Nolan JP, Morley PT, Vanden Hoek TL, Hickey RW, Kloeck WG, Billi J, Bottiger BW, Morley PT, Nolan JP, Okada K, Reyes C, Shuster M, Steen PA, Weil MH, Wenzel V, Hickey RW, Carli P, Vanden Hoek TL, Atkins D, International Liaison Committee on R (2003). Therapeutic hypothermia after cardiac arrest: an advisory statement by the advanced life support task force of the International Liaison Committee on Resuscitation. Circulation.

[65] Limatola V, Ward P, Cattano D, Gu J, Giunta F, Maze M, Ma D (2010). Xenon preconditioning confers neuroprotection regardless of gender in a mouse model of transient middle cerebral artery occlusion. Neuroscience.

[66] Fries M, Nolte KW, Coburn M, Rex S, Timper A, Kottmann K, Siepmann K, Hausler M, Weis J, Rossaint R (2008). Xenon reduces neurohistopathological damage and improves the early neurological deficit after cardiac arrest in pigs. Crit Care Med.

[67] Fries M, Brucken A, Cizen A, Westerkamp M, Lower C, Deike-Glindemann J, Schnorrenberger NK, Rex S, Coburn M, Nolte KW, Weis J, Rossaint R, Derwall M (2012). Combining xenon and mild therapeutic hypothermia preserves neurological function after prolonged cardiac arrest in pigs. Crit Care Med.

[68] Kumar A, Stoica BA, Loane DJ, Yang M, Abulwerdi G, Khan N, Kumar A, Thom SR, Faden AI (2017). Microglial-derived microparticles mediate neuroinflammation after traumatic brain injury. J Neuroinflammation.

[69] Sheng SP, Lei B, James ML, Lascola CD, Venkatraman TN, Jung JY, Maze M, Franks NP, Pearlstein RD, Sheng H, Warner DS (2012). Xenon neuroprotection in experimental stroke: interactions with hypothermia and intracerebral hemorrhage. Anesthesiology.

[70] Chakkarapani E, Dingley J, Liu X, Hoque N, Aquilina K, Porter H, Thoresen M (2010). Xenon enhances hypothermic neuroprotection in asphyxiated newborn pigs. Ann Neurol.

[71] Azzopardi D, Robertson NJ, Bainbridge A, Cady E, Charles-Edwards G, Deierl A, Fagiolo G, Franks NP, Griffiths J, Hajnal J, Juszczak E, Kapetanakis B, Linsell L, Maze M, Omar O, Strohm B, Tusor N, Edwards AD (2016). Moderate hypothermia within 6 h of birth plus inhaled xenon versus moderate hypothermia alone after birth asphyxia (TOBY-Xe): a proof-of-concept, open-label, randomised controlled trial. Lancet Neurol.

[72] Arola OJ, Laitio RM, Roine RO, Gronlund J, Saraste A, Pietila M, Airaksinen J, Perttila J, Scheinin H, Olkkola KT, Maze M, Laitio TT (2013). Feasibility and cardiac safety of inhaled xenon in combination with therapeutic hypothermia following out-of-hospital cardiac arrest. Crit Care Med.

[73] Laitio R, Hynninen M, Arola O, Virtanen S, Parkkola R, Saunavaara J, Roine RO, Gronlund J, Ylikoski E, Wennervirta J, Backlund M, Silvasti P, Nukarinen E, Tiainen M, Saraste A, Pietila M, Airaksinen J, Valanne L, Martola J, Silvennoinen H, Scheinin H, Harjola VP, Niiranen J, Korpi K, Varpula M, Inkinen O, Olkkola KT, Maze M, Vahlberg T, Laitio T (2016). Effect of inhaled xenon on cerebral white matter damage in comatose survivors of out-of-hospital cardiac arrest: a randomized clinical trial. JAMA.

[74] Schmidt M, Marx T, Kotzerke J, Luderwald S, Armbruster S, Topalidis P, Schirmer U, Reinelt H (2001). Cerebral and regional organ perfusion in pigs during xenon anaesthesia. Anaesthesia.

[75] Schmidt M, Marx T, Papp-Jambor C, Schirmer U, Reinelt H (2002). Effect of xenon on cerebral autoregulation in pigs. Anaesthesia.

[76] Plougmann J, Astrup J, Pedersen J, Gyldensted C (1994). Effect of stable xenon inhalation on intracranial pressure during measurement of cerebral blood flow in head injury. J Neurosurg.

[77] Brohan J, Goudra BG (2017). The role of GABA receptor agonists in anesthesia and sedation. CNS Drugs.

[78] Cremer J, Stoppe C, Fahlenkamp AV, Schalte G, Rex S, Rossaint R, Coburn M (2011). Early cognitive function, recovery and well-being after sevoflurane and xenon anaesthesia in the elderly: a double-blinded randomized controlled trial. Med Gas Res.

[79] Campos-Pires R, Hirnet T, Valeo F, Ong BE, Radyushkin K, Aldhoun J, Saville J, Edge CJ, Franks NP, Thal SC, Dickinson R (2019). Xenon improves long-term cognitive function, reduces neuronal loss and chronic neuroinflammation, and improves survival after traumatic brain injury in mice. Br J Anaesth.

[80] Billings FT, Ball SK, Roberts LJ, Pretorius M (2011). Postoperative acute kidney injury is associated with hemoglobinemia and an enhanced oxidative stress response. Free Radic Biol Med.

[81] Yang CW, Li C, Jung JY, Shin SJ, Choi BS, Lim SW, Sun BK, Kim YS, Kim J, Chang YS, Bang BK (2003). Preconditioning with erythropoietin protects against subsequent ischemia-reperfusion injury in rat kidney. FASEB J.

[82] Stevanovic A, Schaefer P, Coburn M, Rossaint R, Stoppe C, Boor P, Pfister D, Heidenreich A, Christ H, Hellmich M, Fahlenkamp AV (2017). Renal function following xenon anesthesia for partial nephrectomy-an explorative analysis of a randomized controlled study. PLoS One.

[83] (2014) Breath it in In: Editor (ed)^(eds) Book Breath it in City, pp.

[84] (2014) Breathe it in. In: Editor (ed)^(eds) Book Breathe it in. City, pp.

[85] Tassel C, Le Dare B, Morel I, Gicquel T (2016). Xenon: from rare gaz to doping product. Presse Med.

[86] Ramos-Cabrer P., Campos F., Sobrino T., Castillo J. (2010). Targeting the Ischemic Penumbra. Stroke.

[87] Helbok R, Shaker E, Beer R, Chemelli A, Sojer M, Sohm F, Broessner G, Lackner P, Beck M, Zangerle A, Pfausler B, Thome C, Schmutzhard E (2012). High dose erythropoietin increases brain tissue oxygen tension in severe vasospasm after subarachnoid hemorrhage. BMC Neurol.

[88] Thevis M, Piper T, Geyer H, Schaefer MS, Schneemann J, Kienbaum P, Schanzer W (2015). Urine analysis concerning xenon for doping control purposes. Rapid Commun Mass Spectrom.

[89] Thevis M, Piper T, Geyer H, Thomas A, Schaefer MS, Kienbaum P, Schanzer W (2014). Measuring xenon in human plasma and blood by gas chromatography/mass spectrometry. Rapid Commun Mass Spectrom.

[90] Schaefer MS, Piper T, Geyer H, Schneemann J, Neukirchen M, Thevis M, Kienbaum P (2017). Xenon elimination kinetics following brief exposure. Drug Test Anal.

[91] Stoppe C, Ney J, Brenke M, Goetzenich A, Emontzpohl C, Schalte G, Grottke O, Moeller M, Rossaint R, Coburn M (2016). Sub-anesthetic xenon increases erythropoietin levels in humans: a randomized controlled trial. Sports Med.

[92] Savitz SI, Baron JC, Fisher M, Consortium SX (2019). Stroke treatment academic industry roundtable X: brain cytoprotection therapies in the reperfusion era. Stroke.

[93] Debevec T, Keramidas ME, Norman B, Gustafsson T, Eiken O, Mekjavic IB (2012). Acute short-term hyperoxia followed by mild hypoxia does not increase EPO production: resolving the “normobaric oxygen paradox”. Eur J Appl Physiol.

[94] Liquide A (2013) Air Liquide launches LENOXe TM. . In: Editor (ed)^(eds) Book Air Liquide launches LENOXe TM. . City, pp.

[95] Roehl AB, Goetzenich A, Rossaint R, Zoremba N, Hein M (2010). A practical rule for optimal flows for xenon anaesthesia in a semi-closed anaesthesia circuit. Eur J Anaesthesiol.

[96] Stoppe C, Rimek A, Rossaint R, Rex S, Stevanovic A, Schalte G, Fahlenkamp A, Czaplik M, Bruells CS, Daviet C, Coburn M (2013). Xenon consumption during general surgery: a retrospective observational study. Med Gas Res.

[97] Horrigan DJ, Wells CH, Guest MM, Hart GB, Goodpasture JE (1979). Tissue gas and blood analyses of human subjects breathing 80% argon and 20% oxygen. Aviat Space Environ Med.

[98] Hollig A, Schug A, Fahlenkamp AV, Rossaint R, Coburn M, Argon Organo-Protective N (2014). Argon: systematic review on neuro- and organoprotective properties of an “inert” gas. Int J Mol Sci.

[99] Ulbrich Felix, Goebel Ulrich (2016). The Molecular Pathway of Argon-Mediated Neuroprotection. International Journal of Molecular Sciences.

[100] Nespoli F, Redaelli S, Ruggeri L, Fumagalli F, Olivari D, Ristagno G (2019). A complete review of preclinical and clinical uses of the noble gas argon: evidence of safety and protection. Ann Card Anaesth.

[101] Loetscher PD, Rossaint J, Rossaint R, Weis J, Fries M, Fahlenkamp A, Ryang YM, Grottke O, Coburn M (2009). Argon: neuroprotection in in vitro models of cerebral ischemia and traumatic brain injury. Crit Care.

[102] Fahlenkamp AV, Rossaint R, Haase H, Al Kassam H, Ryang YM, Beyer C, Coburn M (2012). The noble gas argon modifies extracellular signal-regulated kinase 1/2 signaling in neurons and glial cells. Eur J Pharmacol.

[103] Ulbrich F, Kaufmann KB, Coburn M, Lagreze WA, Roesslein M, Biermann J, Buerkle H, Loop T, Goebel U (2015). Neuroprotective effects of Argon are mediated via an ERK-1/2 dependent regulation of heme-oxygenase-1 in retinal ganglion cells. J Neurochem.

[104] Abraini JH, Kriem B, Balon N, Rostain JC, Risso JJ (2003). Gamma-aminobutyric acid neuropharmacological investigations on narcosis produced by nitrogen, argon, or nitrous oxide. Anesth Analg.

[105] Fahlenkamp AV, Coburn M, de Prada A, Gereitzig N, Beyer C, Haase H, Rossaint R, Gempt J, Ryang YM (2014). Expression analysis following argon treatment in an in vivo model of transient middle cerebral artery occlusion in rats. Med Gas Res.

[106] Zhuang L, Yang T, Zhao H, Fidalgo AR, Vizcaychipi MP, Sanders RD, Yu B, Takata M, Johnson MR, Ma D (2012). The protective profile of argon, helium, and xenon in a model of neonatal asphyxia in rats. Crit Care Med.

[107] Ulbrich F, Kaufmann K, Roesslein M, Wellner F, Auwarter V, Kempf J, Loop T, Buerkle H, Goebel U (2015). Argon mediates anti-apoptotic signaling and neuroprotection via inhibition of Toll-like receptor 2 and 4. PLoS One.

[108] Zhao H, Mitchell S, Ciechanowicz S, Savage S, Wang T, Ji X, Ma D (2016). Argon protects against hypoxic-ischemic brain injury in neonatal rats through activation of nuclear factor (erythroid-derived 2)-like 2. Oncotarget.

[109] Koziakova MH, Katie; Edge, Christopher; Franks, Nicholas; White, Ian; Dickinson, Robert;, (2019) Noble gas neuroprotection: xenon and argon protect against hypoxic-ischemic brain injury in vitro via different mechanisms, while helium, neon and krypton are without effect. Br J Anaesth 2019;123(5):601-609 10.1016/j.bja.2019.07.010 PMC6871267 31470983

[110] Brucken A, Bleilevens C, Fohr P, Nolte K, Rossaint R, Marx G, Fries M, Derwall M (2017). Influence of argon on temperature modulation and neurological outcome in hypothermia treated rats following cardiac arrest. Resuscitation.

[111] Brucken A, Kurnaz P, Bleilevens C, Derwall M, Weis J, Nolte K, Rossaint R, Fries M (2015). Delayed argon administration provides robust protection against cardiac arrest-induced neurological damage. Neurocrit Care.

[112] Ryang YM, Fahlenkamp AV, Rossaint R, Wesp D, Loetscher PD, Beyer C, Coburn M (2011). Neuroprotective effects of argon in an in vivo model of transient middle cerebral artery occlusion in rats. Crit Care Med.

[113] David HN, Haelewyn B, Degoulet M, Colomb DG, Risso JJ, Abraini JH (2012). Ex vivo and in vivo neuroprotection induced by argon when given after an excitotoxic or ischemic insult. PLoS One.

[114] Ma S, Chu D, Li L, Creed JA, Ryang YM, Sheng H, Yang W, Warner DS, Turner DA, Hoffmann U (2019). Argon inhalation for 24 hours after onset of permanent focal cerebral ischemia in rats provides neuroprotection and improves neurologic outcome. Crit Care Med.

[115] Ma Y, Wang J, Wang Y, Yang GY (2017). The biphasic function of microglia in ischemic stroke. Prog Neurobiol.

[116] Ulbrich F, Lerach T, Biermann J, Kaufmann KB, Lagreze WA, Buerkle H, Loop T, Goebel U (2016). Argon mediates protection by interleukin-8 suppression via a TLR2/TLR4/STAT3/NF-kappaB pathway in a model of apoptosis in neuroblastoma cells in vitro and following ischemia-reperfusion injury in rat retina in vivo. J Neurochem.

[117] Liu J, Nolte K, Brook G, Liebenstund L, Weinandy A, Hollig A, Veldeman M, Willuweit A, Langen KJ, Rossaint R, Coburn M (2019). Post-stroke treatment with argon attenuated brain injury, reduced brain inflammation and enhanced M2 microglia/macrophage polarization: a randomized controlled animal study. Crit Care.

[118] Surinkaew P, Sawaddiruk P, Apaijai N, Chattipakorn N, Chattipakorn SC (2018). Role of microglia under cardiac and cerebral ischemia/reperfusion (I/R) injury. Metab Brain Dis.

[119] Franco EC, Cardoso MM, Gouveia A, Pereira A, Gomes-Leal W (2012). Modulation of microglial activation enhances neuroprotection and functional recovery derived from bone marrow mononuclear cell transplantation after cortical ischemia. Neurosci Res.

[120] Irani Y, Pype JL, Martin AR, Chong CF, Daniel L, Gaudart J, Ibrahim Z, Magalon G, Lemaire M, Hardwigsen J (2011). Noble gas (argon and xenon)-saturated cold storage solutions reduce ischemia-reperfusion injury in a rat model of renal transplantation. Nephron Extra.

[121] Faure A, Bruzzese L, Steinberg JG, Jammes Y, Torrents J, Berdah SV, Garnier E, Legris T, Loundou A, Chalopin M, Magalon G, Guieu R, Fenouillet E, Lechevallier E (2016). Effectiveness of pure argon for renal transplant preservation in a preclinical pig model of heterotopic autotransplantation. J Transl Med.

[122] De Deken J, Rex S, Lerut E, Martinet W, Monbaliu D, Pirenne J, Jochmans I (2018). Postconditioning effects of argon or xenon on early graft function in a porcine model of kidney autotransplantation. Br J Surg.

[123] Savary G, Lidouren F, Rambaud J, Kohlhauer M, Hauet T, Bruneval P, Costes B, Cariou A, Ghaleh B, Mongardon N, Tissier R (2018). Argon attenuates multiorgan failure following experimental aortic cross-clamping. Br J Clin Pharmacol.

[124] Grune F, Kazmaier S, Hoeks SE, Stolker RJ, Coburn M, Weyland A (2017). Argon does not affect cerebral circulation or metabolism in male humans. PLoS One.

[125] Weyland A, Stephan H, Kazmaier S, Weyland W, Schorn B, Grune F, Sonntag H (1994). Flow velocity measurements as an index of cerebral blood flow. Validity of transcranial Doppler sonographic monitoring during cardiac surgery. Anesthesiology.

[126] Cucino A, Ruggeri L, Olivari D, De Giorgio D, Latini R, Ristagno G (2019). Safety of ventilation with an argon and oxygen gas mixture. Br J Anaesth.

[127] Suleiman S, Klassen S, Katz I, Balakirski G, Krabbe J, von Stillfried S, Kintsler S, Braunschweig T, Babendreyer A, Spillner J, Kalverkamp S, Schroder T, Moeller M, Coburn M, Uhlig S, Martin C, Rieg AD (2019). Argon reduces the pulmonary vascular tone in rats and humans by GABA-receptor activation. Sci Rep.

[128] Dingley J, Liu X, Gill H, Smit E, Sabir H, Tooley J, Chakkarapani E, Windsor D, Thoresen M (2015). The feasibility of using a portable xenon delivery device to permit earlier xenon ventilation with therapeutic cooling of neonates during ambulance retrieval. Anesth Analg.

[129] Cheung CX, Healy DA, Walsh SR (2016). Remote preconditioning and cardiac surgery: regrouping after Remote Ischemic Preconditioning for Heart Surgery (RIPHeart) and Effect of Remote Ischemic Preconditioning on Clinical Outcomes in Patients Undergoing Coronary Artery Bypass Surgery (ERICCA). J Thorac Dis.

[130] Ulbrich F, Eisert L, Buerkle H, Goebel U, Schallner N (2016). Propofol, but not ketamine or midazolam, exerts neuroprotection after ischaemic injury by inhibition of Toll-like receptor 4 and nuclear factor kappa-light-chain-enhancer of activated B-cell signalling: a combined in vitro and animal study. Eur J Anaesthesiol.

[131] Zhang Y, Shan GJ, Zhang YX, Cao SJ, Zhu SN, Li HJ, Ma D, Wang DX, First Study of Perioperative Organ Protection i (2018). Propofol compared with sevoflurane general anaesthesia is associated with decreased delayed neurocognitive recovery in older adults. Br J Anaesth.

[132] Li Volti G, Avola R, Tibullo D (2017). Editorial - propofol as an intraoperative strategy for organ protection. Eur Rev Med Pharmacol Sci.

[133] Cho Youn, Nam Karam, Kim Tae, Choi Seong, Kim Sung, Hausenloy Derek, Jeon Yunseok (2019). Sevoflurane, Propofol and Carvedilol Block Myocardial Protection by Limb Remote Ischemic Preconditioning. International Journal of Molecular Sciences.

[134] Cucino A., Ruggeri L., Olivari D., De Giorgio D., Latini R., Ristagno G. (2019). Safety of ventilation with an argon and oxygen gas mixture. British Journal of Anaesthesia.

[135] Thomas A, Detilleux J, Flecknell P, Sandersen C (2017). Impact of Stroke Therapy Academic Industry Roundtable (STAIR) guidelines on peri-anesthesia care for rat models of stroke: a meta-analysis comparing the years 2005 and 2015. PLoS One.

[136] Campos-Pires R, Armstrong SP, Sebastiani A, Luh C, Gruss M, Radyushkin K, Hirnet T, Werner C, Engelhard K, Franks NP, Thal SC, Dickinson R (2015). Xenon improves neurologic outcome and reduces secondary injury following trauma in an in vivo model of traumatic brain injury. Crit Care Med.

[137] Ma D, Hossain M, Chow A, Arshad M, Battson RM, Sanders RD, Mehmet H, Edwards AD, Franks NP, Maze M (2005). Xenon and hypothermia combine to provide neuroprotection from neonatal asphyxia. Ann Neurol.

